# Exhaustion Pseudo-Paresis

**Published:** 1918-10

**Authors:** 


***Exhaustion Pseudo-Paresis*; *a Fatigue Syndrome Simulating Early Paresis Developing Under Intensive Military Training*.** By J. Ramsay Hunt, *The Journal of Nervous and Mental Diseases,* March 1918.

The author presents a résumé of the results of special neuropsychiatric examination in one of the large training camps for officers.

Generally speaking, the clinical symptoms were not very marked. General cerebral symptoms or evidence of mental deterioration were so slight as to be practically negligible.

As directly bearing on this subject, emphasis was laid on the occurrence of another group of cases because of the close similarity of the somatic symptoms to those of early paresis. The pupils in all of the cases were unequal and showed a very feeble and sluggish reaction to light. In some of the cases there was so little response as to suggest a true loss of light reaction. The reaction on accommodation was preserved and the sympathetic response was also elicitable.

Clinically, the diagnosis of incipient paresis seemed assured, but the serological examinations of the blood and spinal fluid were entirely negative. There was no increase of globulin or cells and the colloidal gold reaction was negative.

Among the recognized mental symptoms of fatigue were a diminished power of attention, a lack of the ability to concentrate, a slow reaction to mental stimuli, slowness in reasoning, as well as errors and slowness in mathematical calculation.

The suspicious pupillary phenomena observed in these exhaustion cases were possibly dependent upon a disturbance of the sympathetic innervation of the pupil by fatigue or the toxins of fatigue.

Dr. Hunt thought that the formal recognition of a fatigue syndrome simulating early paresis was worthy of earnest consideration, and it was not unlikely that under still greater conditions of stress and strain this group of cases might be the forerunner of more severe types of the exhaustion neuroses and neuropsychoses.

